# Comparing the Long-Term Success Rates of Tooth Preservation and Dental Implants: A Critical Review

**DOI:** 10.3390/jfb14030142

**Published:** 2023-03-03

**Authors:** Suelen Cristina Sartoretto, Jamil Awad Shibli, Kayvon Javid, Khalila Cotrim, Antonio Canabarro, Rafael Seabra Louro, Adam Lowenstein, Carlos Fernando Mourão, Vittorio Moraschini

**Affiliations:** 1 Department of Oral Surgery, School of Dentistry, Fluminense Federal University, Niterói 24020-140, Brazil; 2 Department of Periodontology and Oral Implantology, Dental Research Division, University of Guarulhos, São Paulo 07023-070, Brazil; 3 Department of Periodontology, Dental Research Division, School of Dentistry, Veiga de Almeida University, Rio de Janeiro 20271-020, Brazil; 4 Division of Periodontology, School of Dentistry, Rio de Janeiro State University, Rio de Janeiro 20551-030, Brazil; 5 Department of Periodontology, Division Dental Research Administration, Tufts University School of Dental Medicine, Boston, MA 02111, USA

**Keywords:** dental implants, endodontic treatment, periodontal treatment, survival analysis, success rates

## Abstract

Implant therapy is considered a predictable, safe, and reliable rehabilitation method for edentulous patients in most clinical scenarios. Thus, there is a growing trend in the indications for implants, which seems attributable not only to their clinical success but also to arguments such as a more “simplified approach” based on convenience or the belief that dental implants are as good as natural teeth. Therefore, the objective of this critical literature review of observational studies was to discuss the evidence concerning the long-term survival rates and treatment outcomes, comparing endodontically or periodontally treated teeth with dental implants. Altogether, the evidence suggests that the decision between keeping a tooth or replacing it with an implant should carefully consider the condition of the tooth (e.g., amount of remaining tooth and degree of attachment loss and mobility), systemic disorders, and patient preference. Although observational studies revealed high success rates and long-term survival of dental implants, failures and complications are common. For this reason, attempts should be made to first save maintainable teeth over the long-term, instead of immediately replacing teeth with dental implants.

## 1. Introduction

Dental implants have been widely used in recent decades to treat edentulous areas or replace extracted teeth [1,2,3,4,5]. Overall, the global market for dental implants was valued at $4.12 billion in 2021, with over 9 million implants placed. With excellent success rates reported, implant therapy is considered a predictable, safe, and reliable rehabilitation method for edentulous patients in most clinical scenarios [5,6,7,8,9,10,11,12]. However, there is a growing trend of increase in the indications for implants, attributable to their clinical success and/or their popularity [7].

These arguments can result in a “simplified approach”, based on convenience, ease, or the tendency to believe that dental implants are as good as natural teeth, rather than an accurate analysis of treatment possibilities and prognosis, leading to the condemnation of teeth that might be rehabilitated [13].

From these considerations, there is a routine dilemma in clinical dentistry that derives from the question: “Should we maintain/restore a compromised tooth or extract it and replace it with an implant-supported restoration?” However, few articles provide the ideal clinical answer based on scientific evidence [14,15]. The primary goals of periodontal therapy are to keep natural teeth functional by maintaining, supporting, or generating the periodontium and, when possible, providing pleasing aesthetics [16,17,18]. Prosthetic alternatives, even if dental implants are supported, cannot compete with natural dentition in their biomechanical, sensory, proprioceptive, and adaptive aspects. Nonetheless, the attempt to maintain a tooth that is in pathological condition can have unfavorable consequences, ranging from loss of function, progressive bone loss, and the extension of odontogenic infections to deep fascial spaces [19].

Several studies have systematically compared the long-term predictability of dental implants to other treatment modalities, such as supportive periodontal therapy or root canal treatment and restoration. However, the success criteria adopted by the authors vary significantly between treatment modalities, making it difficult to compare success rates directly [13,20]. Additionally, the outcome is affected by various uncountable factors, such as patient adherence to treatment, parafunctional habits, systemic condition, smoking status, and clinician experience [1,21,22,23,24].

**Figure 1:**
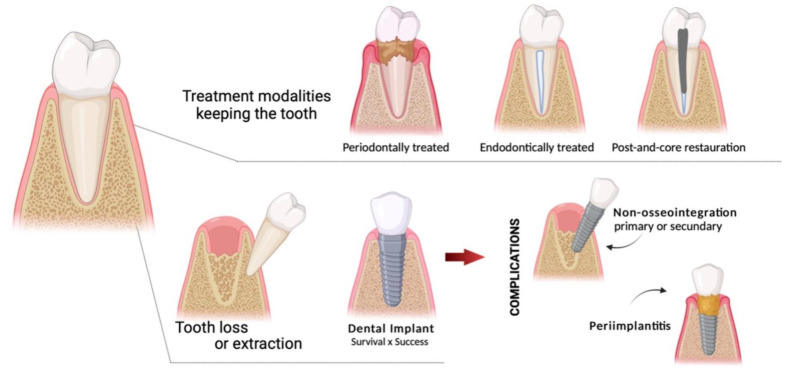
Graphical abstract showing the possible evolutions between treating or extracting a tooth. This figure was created with Biorender.com.

To provide insight into the clinical decision on the maintenance of the dental element and its treatment possibilities or replacement with an osseointegrated implant, we performed a critical literature review to discuss the long-term survival rates and treatment outcomes, comparing endodontically or periodontally treated teeth and dental implants (Figure 1).

## 2. Definitions and Search Process

Most longitudinal studies adopt the concept of survival rate to monitor the outcomes of treatments in implants and teeth. For the present critical review, the presence of the implant or tooth in the oral cavity was considered as survival. In order to survey the current state of evidence, this critical review conducted a narrative review of the literature in the main databases for clinical (randomized clinical trials) and observational studies (prospective, retrospective, cross-sectional, and case controls).

## 3. Survival of Endodontically Treated Teeth

Contemporary dentistry aims to preserve and restore natural teeth [25]. However, the continuous controversy in this field is whether endodontic treatment associated with a restoration may overcome the long-term success of an osseointegrated dental implant [25,26].

Prevailing dentistry trends seem to indicate a “simplified approach”, favoring placing implants as a standard treatment that is more convenient than the endodontic approach. Meanwhile, due to the difficulty of standardization to establish less biased comparisons, randomized clinical studies that compare the outcomes of nonsurgical root canal treatment (NRCT) and single-tooth implants (SI) to dictate the correct selection of modality remain absent [26,27].

This limitation in comparing the survival of treatments is related to the considerable variability among study designs and, mainly, to the definition of survival [28]. The most reported criterion for implant survival is osseointegration [29]. This outcome differs from those of endodontic treatments, which usually include the function, clinical signs, symptomatology, and radiographic healing related to the periapical status. Importantly, even if a tooth is fully functional at the time of reevaluation, endodontic treatment may be considered unsatisfactory if deficient healing is detected in an X-ray [30,31].

While an evidence-based decision tree should guide the choice of endodontic treatment or extraction, followed by dental implants, this question has not been satisfactorily answered [32]. Despite the difficulty of comparison, the decision to treat a tooth endodontically or replace it with an implant must be based on tooth-related clinical and radiographic conditions other than the outcomes of the procedures themselves [32].

An end-stage tooth is a structural deficiency or a pathologic situation that cannot be successfully repaired by endodontic treatment. The tooth continues to exhibit progressive pathologic changes and clinical dysfunction [27]. This concept, associated with the best available evidence and the patient’s desires and needs, should be considered, and factored prior to deciding between endodontic therapy and dental implants [27].

A recent retrospective study compared the outcomes of NRCT and SI, evaluating 3671 patients who received at least 1 NRCT and 1 SI. The results demonstrated a 95% survival rate (7.5-year follow-up) for both treatments [31]. These results corroborate other authors who, despite presenting different survival rates (83.34% for NRCT and 80.8% for IS), show no significant differences among the treatments [26]. Notably, the costs of the procedure, number of additional treatments, number of appointments, elapsed time before the final restoration, and number of prescribed medications were significantly higher for SI than for NRCT [31]. Furthermore, sensory, proprioceptive, and adaptive aspects of maintaining natural dentition are favored.

Over the past few decades, considerable advances have been made in endodontic practices, which, in some instances, has enabled the preservation of teeth that, in the past, would have been extracted [26,27]. One of these advances is endodontic microsurgery, which is a technique that is applied when the tooth has not healed through NRCT. The outcomes of this modality for tooth preservation were also recently compared to SI in a systematic review. The survival rates for SI and endodontic microsurgery were both high, yet, in this case, they were higher for single implants. Nevertheless, the different criteria established to evaluate the survival of these modalities of treatment limit direct comparisons [25].

A series of studies evaluated the survival and success rates of nonsurgical root canal treatment in the literature. Among them, a systematic review included 14 studies published between 1993 and 2007. The reported percentages of tooth survival ranged from 86% to 93% [30]. A 20-year historical prospective cohort study evaluated the number of healthy roots filled following manual canal instrumentation. The evaluation included 79 patients—196 teeth—of which, 20.9% were extracted for non-endodontic reasons, while 79.1% survived. Of the teeth that survived, 4.5% were characterized as symptomatic retreatment teeth, 65% were healthy teeth, and 9.1% were asymptomatic functional teeth. Only two teeth were extracted for endodontic reasons [33].

Scientific evidence based on retrospective studies investigating 10-year survival rates of NRCT presented similar results. Boren et al. (2014) [34] evaluated 420 teeth treated in a public endodontic specialty clinic. The survival was 81.5%, of which 17.4% teeth were extracted, of which 6.8% were, in turn, related to endodontic diagnoses. Fernandez et al. 2017 [35] observed a survival rate of more than 90% in a study of 132 teeth.

The main factors that are reported as significantly prognostic for the success of NRCT are the presence of periapical lesions before treatment, isolation of the operative field, density/extent of root canal filling, and coronal seal quality [35,36,37]. Additionally, age, tooth type, presence of mesial/distal contacts, nonfunctioning as an abutment for fixed or removable prostheses, and being restored with a crown after NRCT were significant prognostic factors for tooth retention [30].

In summary, an endodontically treated tooth or a dental implant does not present a lifetime guarantee. Both possibilities should be comprehended as complementing each other and not as competing. These therapies should serve the overall goals in dentistry, namely showing long-term success and benefit to the patient, being the least invasive options, and combining function, satisfaction, and aesthetics [28]. To obtain these purposes, it is essential for dentists to understand and value the long-term outcomes of both implants and endodontically treated teeth and work as a collaborative team comprising dentists and specialists to best serve patients.

## 4. Survival of Teeth Treated with Post-and-Core Restorations

The coronal destruction induced by caries, fractures, non-carious lesions, or previous restorative treatments. access preparation. and the percentage of structural integrity loss during root canal treatment following the mechanical procedures of endodontic therapies all affect a tooth’s capacity to resist functional and parafunctional forces [38].

Long-term survival rates of restorations following standard endodontic treatment range between approximately 81% and 100% [32]. However, endodontically restored teeth are more prone to failure and fracture than vital teeth [38]. Thus, teeth with severe damage, considered vulnerable and more susceptible to fracture than vital teeth, may benefit from prosthetic restorations by intraradicular retainers when the remnant tooth structure is no longer sufficient [39,40].

Different materials have been used for intracanal retainers to favorably increase the number of endodontically treated teeth and restoration survival. In general, cast (metal) post-and-core, such as gold [41], stainless steel [42], and titanium [43], is used to retain single or multiple total prosthetic crowns. Another category of less rigid materials, such as prefabricated glass fiber posts retained through adhesive cementing in the root canals and custom glass fiber posts, have also been utilized, with varying degrees of success [44,45].

A series of conditions must be considered when deciding which material to utilize for intraradicular retainers. The material’s elastic modulus, when most similar to dentinal structures, promotes a uniform distribution of stress on the long tooth axis, decreasing the risk of failures [46,47]. Furthermore, the parafunctional habits of the patient, the aesthetic demand, and the region of the rehabilitated tooth are also factors to be considered [48].

The literature presents differences in reported failure rates between fiber and metal posts in endodontically treated teeth. While fiber posts are utilized more frequently, post debonding, loss of retention of single crowns, and marginal gaps [49] are commonly reported issues. Cast post-and-core can evolve with more severe complications, such as root fractures. However, it appears that the strength and thickness of the remaining dental tooth structure are more critical than the post-material, design, and/or cement material utilized [50]. This condition may significantly interfere with the long-term success rates of post-and-core restorations covered by a crown [51,52].

The high complexity during endodontically treated teeth with post-and-core procedures associated with substantial reduction of the tooth structure may demonstrate increasing complication and failure rates of crowned teeth [53]. Therefore, fixed prosthodontics therapy with dental implants may be considered a better therapeutic alternative in cases where more extensive damage to the remaining teeth is noted. Nevertheless, this fine balance (how much tooth structure loss is too much) often leads to an inappropriate indication for tooth extraction, plus SI, and has resulted in the sacrifice of many savable teeth [7]. In this way, this increasingly frequent dilemma in dentistry, that is, when to retain/restore a compromised tooth versus when to extract it and replace it with a dental implant, needs further review and research [7].

It is also essential to keep in mind that the primary purpose of periodontal therapy is to maintain natural teeth in good function with satisfactory aesthetics. Additionally, the biochemical and sensorial properties of a natural tooth, including its proprioception [54] and adaptation under mechanical forces mediated by the periodontal ligament [55], are some of the main advantages over dental implants.

Direct comparisons between osseointegrated implants and unitary dental rehabilitation techniques using post-and-core restorations are scarce in the literature. The many variables associated with each therapy, such as different treatment steps, variations in the materials used, and the clinician’s ability, promote a great deal of difficulty regarding the standardization requirements to properly conduct proper systematic reviews of randomized clinical studies with less biased comparisons. In this context, information regarding the long-term success rates with more modern materials and techniques using post-and-core restorations may clarify these clinical decisions in the future.

Naumann et al. (2018) [52] performed a systematic review (SR) involving randomized and prospective clinical trials comparing the impact of post versus no-post placement on tooth and restoration survival in ferruled and/or unferruled teeth over a 5+ year time period. The authors highlight the importance of the remaining coronal tooth structure as a predictive factor for both restoration and tooth survival. Similar results were reported by an SR [56] comparing the clinical performance of teeth restored with post-and-core restorations in a follow-up ranging from 6 months to 10 years. While metal and fiber posts present similar clinical behaviors at short- to medium-term follow-up, the remaining dental structure and ferrule significantly increased the survival of restored pulpless teeth. Nevertheless, in the basic evaluation of the therapeutic value of post-and-core treatments, survival time is an important parameter [41]. More long-term clinical studies are essential for understanding the survival rates of the post-and-core restorations under various clinical settings.

A long-term clinical follow-up study (17-year survival) evaluated different metal post-and-core restorations with a covering crown [42]. In this study, 307 core restorations were analyzed. The teeth were assessed to have substantial dentin height or minimal dentin height. The post-and-core restorations under investigation were cast post-and-core restorations, prefabricated metal post and resin composite core restorations, and post-free all-composite core restorations. This study demonstrated no difference in survival probabilities among different core restorations under a covering crown of endodontically treated teeth. The survival rates at the restoration level varied from 71% to 80% and at the tooth level from 83% to 92%. The preservation of a large remaining coronal tooth structure appeared to be most critical toward the long-term survival of endodontically treated crowned teeth.

Balkenhol et al. (2007) [41] performed a 10-year retrospective longitudinal study evaluating the survival time of custom-fabricated cast posts and cores and possible covariates, which affected the risk of failure. The average survival time of all 802 posts and cores was 7.3 years, and failures were recorded in 90 cases (11.2%) at the final examination time. However, this did not necessarily involve a new prosthetic restoration. The most common cause of failure was the loss of retention of the post-and-core (43.3%). Posts and cores fabricated from high-gold-content alloys had a significantly higher survival probability than posts fabricated from a semiprecious alloy (log-rank test *p* < 0.01). Directly fabricated posts and cores exhibited a lower survival probability than indirectly fabricated posts (log-rank test *p* < 0.01).

As previously cited, the rehabilitated tooth’s anatomic region is an important point to consider in a prosthetic restoration. Although several articles report a significant number of failures in anterior teeth, when compared to posterior teeth (this difference being partially explained by the greater horizontal forces presented in the anterior region [50]), some studies indicate a higher frequency of fractures in posterior teeth. This has been reported to be due to the absence of remaining walls on the treated teeth [57,58]. A recent systematic review evaluated RCTs comparing the failure rates of anterior and posterior teeth treated with post-and-core restorations [59]. The failure rate of teeth was the primary outcome of the study, and different types of posts were included. No difference between anterior and posterior teeth treated with post-and-core restorations was identified. Additionally, no difference was found between incisors and canines, and premolars showed no statistically significant difference when compared to molars.

One finding relevant to this article is that the comparison of endodontically treated teeth with and without post-and-core restorations should not be gathered into studies and compared to dental implant survival rates. The technique using post-and-core restorations, including a larger number of clinical and laboratory steps than normal endodontic therapy, generally results in a much higher failure rate. In summary, a fundamental point in the literature that determines the highest survival rate of post-and-core restorations is the remaining dental tooth structure, such as that of ferruled teeth. It is imperative to comprehend the scientific evidence regarding the different techniques and their biological concepts, including the critical assessment of long-term studies.

## 5. Survival of Periodontal Treated Teeth 

Periodontal disease is a bacterial biofilm caused by chronic inflammatory disease resulting in destruction of periodontal supporting tissues and pocket formation [60,61,62,63,64,65]. When left untreated or not adequately managed, the progressive destruction of the periodontal tissues may ultimately result in tooth loss, and it is nowadays considered the primary cause of tooth loss worldwide [66,67]. On the other hand, when appropriate treatment is provided, periodontally treated teeth can function for years at limited cost, even in the presence of progressive bone loss [68,69]. Thus, the goal of periodontal therapy is to preserve, improve, and maintain the natural dentition ensuring function and aesthetics.

The first steps (i.e., Step 1 and 2) of periodontal therapy consist of reducing or eliminating pathogenic (disease-associated) biofilm through self-performed oral hygiene measures and supra- and subgingival mechanical debridement [17,70]. 

After the treatment of active disease, supportive periodontal therapy (SPT) begins, and patients are routinely closely monitored to ensure that any colonizing microbial populations in residual periodontal pockets are removed [18,71]. These procedures aim to avoid the recurrence and progression of periodontal disease and prevent or reduce tooth loss incidence [72]. Several long-term, retrospective, epidemiological studies have confirmed the success of SPT and demonstrated that only 2% to 5% of teeth in patients originally treated for chronic periodontitis are lost over a 5- to 10-year period [73,74,75,76,77].

It has been demonstrated that some teeth respond better to periodontal therapy than others [78]. Notably, molar furcation sites respond less favorably to periodontal therapy than non-molar sites and molar flat-surface sites of similar probing depth. These findings are compatible with other authors, who have observed that multi-rooted teeth show a less favorable probing pocket depth reduction than single-rooted teeth. Various authors [79,80] have suggested that local anatomical conditions of multi-rooted teeth show difficulties in the treatment of periodontal infection.

Periodontal teeth with advanced bone loss are often mobile, impacting their masticatory and phonetic functionality and reducing patients’ comfort. This mobility can be decreased by splinting (connecting these teeth to their adjacent neighbors) [81]. Graetz et al. [82] have shown that splinting does not negatively affect the prognosis of splinted teeth. They concluded that long-term tooth survival of splinted teeth was possible in compliant patients with periodontitis, and teeth with high mobility can be successfully splinted to stabilize them. However, splint repair is frequently needed.

As previously mentioned, several studies have demonstrated how crucial effective periodontal treatment and long-term SPT are to prevent tooth loss [14,74,83] and long-term tooth survival [82]. The decision between retaining a compromised tooth or extracting the tooth and placing a dental implant requires adequate training and evidence-based judgment. Levin and Halperin-Sternfeld [13] conducted a systematic review of the long-term survival rates of teeth and implants. They suggested that, even when a tooth might seem compromised and require treatment to be maintained, it should be kept in mind that implant treatment also requires surgery and additional costs and often requires bone augmentation and additional procedures that are not free of risk.

It is obvious that treatments with dental substitutes can be considered an option, especially for missing teeth and in severe cases of periodontal diseases. However, it would be important that more efforts be made to treat periodontal diseases, focusing on what truly works (long-term biofilm self-control and motivation), mainly because it does not seem valid to offer treatment alternatives, such as dental implants, that are also harmed by the ubiquitous presence of biofilms.

Undoubtedly, novel techniques and technologies in the field of reconstructive dentistry have potential applications to satisfy the specific needs of the patient [84], but as stated by Pjetursson et al. [85], implants are supposed to replace missing teeth; they are not supposed to replace savable teeth.

## 6. Survival of Dental Implants 

The number of edentulous patients rehabilitated through dental implants increases exponentially each year [86]. Currently, there is a strong tendency to “extract and place an implant”. However, this decision is complex and must consider several parameters. With the increase in the number of implants placed each year, the number of failures and complications related to treatments has also been increasing [87]. Implant rehabilitations require a surgical and prosthetic learning curve. Otherwise, biological, or mechanical complications are expected [88,89,90,91,92,93,94,95].

Decision making in modern dentistry should be based on scientific evidence. However, the decision between toot retention or extraction should consider parameters such as the masticatory function, the systemic condition of the patient, the conditions of the periodontium, and the cost of treatment. In addition, it is essential that the decision is shared with the patient’s expectations.

Modern implant dentistry has numerous benefits when compared with previous treatment modalities. Implants with a bioactive surface induce faster osseointegration [2]. In addition, increasingly less invasive and more accurate surgical techniques make treatments faster, less morbid, and more predictable [3]. This makes implants an important tool for the rehabilitation of missing teeth. For example, the use of implants for the rehabilitation of unitary losses reduces the need for the indication of fixed partial dentures (FDPs) and, consequently, the need to prepare adjacent teeth with healthy dental structures. Additionally, the survival of single implants is usually superior to that of FDPs [20], especially when one of the abutments has been endodontically treated [96].

Clinicians often must decide between maintaining or extracting a tooth with a doubtful prognosis. This decision can be difficult and complex. The use of preestablished criteria can assist in this process, such as the criteria proposed by Strindberg (1956) [97] and Ørstavik et al. (1986) [98] for endodontically treated teeth and parameters, such as pocket depth, degree of mobility, and bleeding on probing for teeth after periodontal treatment [16,99]. In this way, it is essential that a correct “endpoint” is determined, so that a treatment can be considered successful. A recent publication observed that a clinical endpoint of ≤4 sites with PD ≥ 5 mm is effective in determining disease remission/control after active periodontal treatment [100].

Implants are currently classified according to their survival and success rates [101]. Survival classification is quantitative and based on whether the implant is present in the oral cavity, independent of implant health. In contrast, the success analysis is qualitative and involves several parameters (e.g., marginal bone loss, probing depth, pain, suppuration, and mobility) that are dependent on the scale adopted [102,103]. Most longitudinal studies report data on survival [104]. This can be justified by the greater practicality in obtaining survival data than in obtaining data on the success rate.

**Figure 2:**
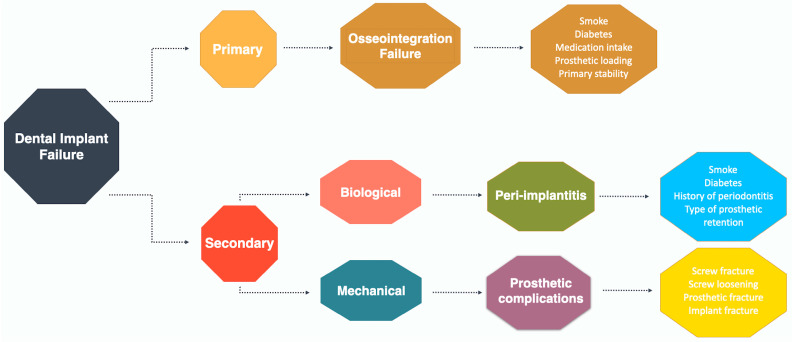
Possible failures of dental implants. Normally, primary failures are related to the osseointegration process, while secondary failures can be of biological or mechanical origin.

There is a logical tendency for implants to present a survival rate greater than the success rate. Moraschini et al. [4] observed long-term cumulative rates of 94.6% and 89.7% for survival and success rates, respectively, after a mean follow-up of 13.4 years. Table 1 and Table 2 reports implant success and survival rates across longitudinal studies with more than 10 years of follow-up. There was a variation in implant survival from 73.4% to 100% at an average of 14.5 years of follow-up. Failures can occur early, related to non-osseointegration of implants (primary), or late, characterized by biological or mechanical complications (secondary) (Figure 2).

A greater number of failures is related to implants supporting FDPs than to single implants [105]. Additionally, single-stage implants have less survival than two-stage implants [106]. However, there is no evidence to date on the length, diameter, and shape of implants, in relation to survival and success rates.

Short implants (≤8 mm) are reported to have survival rates, marginal bone loss, and prosthetic complications equivalent to those of long implants [107]. A recent systematic review [108] evaluated the effectiveness of extra-short implants (5 and 6 mm in length). It was concluded that extra-short implants are feasible in ridges exhibiting atrophy, demonstrating satisfactory survival rates, as well as low rates of prosthetic and biologic complications, across a 5-year follow-up. In this way, the use of short implants can be an interesting option as an alternative to surgery for bone reconstruction.

In general, dental implants have a high survival (94.6%) rate after a mean period of 13.4 years of follow-up [4], with the main biological and mechanical complications being mucositis and loss of the prosthetic screw, respectively [4,109].

## 7. Implications for Clinical Practice and Future Perspective 

The passage highlights the importance of investing in tooth maintenance, based on the results of longitudinal studies. The authors caution that the simple replacement of a tooth with a dental implant may not always be successful in the medium- and long-term, particularly for patients who have lost teeth due to periodontal disease. The authors note that such patients may have a higher risk of developing peri-implantitis [87,110], emphasizing the importance of a correct diagnosis and considering the systemic conditions of the patients in treatment planning.

Furthermore, the authors stress the need for future long-term longitudinal studies to evaluate the behavior of rehabilitative treatments for periodontal disease involving prosthetically reconstructed teeth and teeth with endodontic lesions. These studies will provide clinicians with valuable information on the efficacy of different treatment options for these conditions, ultimately improving patient outcomes.

## 8. Conclusions

- The available evidence indicates similar long-term survival rates between endodontically treated teeth and dental implants.
- Regarding teeth treated with post-and-core restorations, the evidence suggests that decision-making to restore a tooth should be based on the amount of remaining tooth structure. This factor is usually more significant than the type of material used for post-and-core buildups.
- The long-term prognosis of teeth treated in the presence of periodontal disease is proportional to the disease stage, quality of treatment, biofilm control, and periodic maintenance.
- Longitudinal studies show high success rates and long-term survival of dental implants. However, failures and complications are common.
- Overall, the evidence suggests that the decision between keeping a tooth or replacing it with an implant should be based on the condition of the tooth (e.g., amount of remaining tooth and degree of attachment loss and mobility), the systemic condition, and patient preference.
- Better attempts should be focused first on saving manageable teeth over the long-term, instead of immediately applying implant therapies.

## Figures and Tables

**Table 1:** Study design follow-up time primary object of the study; number of participants gender dropout (%); age range mean age; number of implants/implant system and implant size (mm) and dental prothesis reported by longitudinal studies with more than 10 years of follow-up.

Author (Year)	Study Design Follow-Up Time Primary Object of the Study	No. of Participants Gender Dropouts (%)	Age Range Mean Age	No. of Implants Implant System Implant Size (mm)	Dental Prosthesis
Lekholm et al. (1999)	Prospective 10 years Implant survival	127 ♂54/♀73 30	18–70 50	461 NobelBiocare ∅7, 10, 13, 15, 18, 20 × 3.75–4.0	FPD
Carlsson et al. (2000)	Prospective 15 years Bone level alteration	60 ♂16/♀44 5	33–64 NR	348 NobelBiocare ∅10–NR	FCDP
Van Steenberghe et al. (2001)	Retrospective 12 years Bone level alteration	158 ♂114/♀44 2.5	32–82 59.2	316 NobelBiocare ∅7, 8, 10, 12, 13, 15, 18, 20 × 3.75, 4.0, 5.0	IOD
Leonhardt et al. (2002)	Prospective 10 years Others	15 ♂8/♀7 21	21–71 NR	57 NobelBiocare ∅NR	FPD
Karoussis et al. (2004)	Prospective 12 years Survival and success	89 ♂34/♀55 29.9	19–79 49.3	179 ITI ∅NR	SC/FPD
Telleman et al. (2006)	Retrospective 10 years Others	38 ♂8/♀30 36.6	46–90 64	115 ITI ∅NR	IOD
Jemt and Johansson (2006)	Retrospective 15 years Others	76 ♂48/♀28 56.6	32–76 61.1	450 NobelBiocare ∅7, 10, 13, 15, 18 × NR	FCDP
Romeo et al. (2006)	Retrospective 14 years Others	129 ♂61/♀68 17.8	NR 53	265 ITI ∅8, 10 × 3.75, 4.1, 4.8	SC/FPD
Åstrand et al. (2008)	Retrospective 20 years Implant survival	21 ♂7/♀14 56.2	40–74 54.3	123 NobelBiocare ∅NR	FCDP
Jemt (2008)	RCT 15 years Others	114 ♂74/♀40 44	NR 42.7	123 NobelBiocare ∅NR	SC
Pikner et al. (2009)	Retrospective 20 years Bone level alteration	640 ♂255/♀385 NR	18–83 52.3	3.462 NobelBiocare ∅NR	SC/FPD/FCDP
Simonis et al. (2010)	Retrospective 16 years Survival and success	55 ♂21/♀34 28	29–88 68.7	131 ITI ∅6, 8, 10, 12 × NR	SC/FPD
Jacobs et al. (2010)	RCT 16 years Others	18 ♂6/♀12 33.3	32–75 55.1	95 NobelBiocare/Astra Tech ∅7, 8, 9, 10, 11, 13, 15, 18, 19 × 3.75, 4.0	FPD
Ma et al. (2010)	RCT 10 years Bone level alteration	106 ♂40/♀66 25.4	NR 65.3	212 NobelBiocare/Southern Implants/Steri-Oss ∅NR	IOD
Mertens et al. (2012)	Prospective 10 years Others	14 ♂3/♀11 14.2	37–71 57.9	52 Astra Tech ∅8, 9 × 3.5, 4.0, 4.5	SC/FPD/FCDP
Lops et al. (2012)	Retrospective 20 years Others	121 ♂57/♀64 24.7	22–69 54	257 ITI ∅8, 10 × 3.75, 4.1, 4.8	SC/FPD/FCDP
Gotfredsen (2012)	Prospective 10 years Others	20 ♂10/♀10 5	18–59 33	20 Astra Tech ∅11, 13, 15 × 4.5	SC
Degidi et al. (2012)	Prospective 10 years Others	48 ♂21/♀27 18.6	NR 49.9	158 NobelBiocare ∅10 to 15 × 3.3, 3.75, 4.0	SC/FPD/FCDP
Deporter et al. (2012)	Prospective 10 years Survival and success	24 ♂8/♀16 20.8	20–72 NR	48 Sybron Implants Solution ∅7, 9 × 4.1	SC/FPD
Deporter et al. (2014)	Prospective 20 years Others	52 ♂17/♀35 32.7	NR 55.3	156 Sybron Implants Solution ∅7, 8, 9, 10 × NR	IOD
Ravald et al. (2013)	RCT 15 years Implant survival	46 ♂27/♀19 25.3	51–88 74.4	371 Astra Tech/NobelBiocare ∅9 to 19 × 3.5, 3.75, 4.0	FCDP
Rocci et al. (2012)	Retrospective 10 years Others	46 ♂26/♀20 NR	24–77 51	97 NobelBiocare ∅8.5 to 18 × NR	SC/FPD
Mangano et al. (2014)	Prospective 10 years Others	194 ♂104/♀90 25.7	24–74 49.1	215 Leone Implant System ∅8 × 3.3, 4.1, 4.8	SC
Adler et al. (2019)	Retrospective 11 years Implant survival	376 ♂207/♀169 NR	20–81 54	1095 Astra Tech/NobelBiocare/Straumann ∅ < 10 and ≥10 × NR	SC/FPD/FCDP

NR, not related; RCT, randomized controlled trial; ♂, men; ♀, women; mm, millimeters; ∅, size; SC, single crowns; FPD, fixed partial dentures; IOD, implant overdenture; FCDP, fixed complete dental prosthesis; %, percentage.

**Table 2:** Mean probing depth (mm); mean marginal bone loss (mm); success rate (%)/criterion of success and survival rate (%) reported by longitudinal studies with more than 10 years of follow-up.

Author (Year)	MPS (mm)	MPOM (mm)	Success Rate (%) Criterion of Success	Survival Rate (%)
Lekholm et al. (1999)	NR	0.7	NR Albrektsson et al. (1986)	92.6
Carlsson et al. (2000)	NR	0.5	99 Albrektsson et al. (1986)	96
Van Steenberghe et al. (2001)	NR	2.67	97.2 Albrektsson et al. (1986)	98.5
Leonhardt et al. (2002)	1.9	1.7	NR	94.7
Karoussis et al. (2004)	2.87	0.98	85.5 Karoussis et al. (2003)	92.4
Telleman et al. (2006)	3.3	2.2	92.2 Albrektsson et al. (1986)	96.3
Jemt and Johansson (2006)	NR	2.1	86.8 Albrektsson et al. (1986)	90.9
Romeo et al. (2006)	2.2	1.65	NR Zarb and Albrektsson (1998) Roos et al. (1997)	97.5
Åstrand et al. (2008)	3.4	2.33	NR	99.2
Jemt (2008)	NR	2	NR Albrektsson and Isidor (1993)	97.7
Pikner et al. (2009)	NR	2.5	NR	98.2
Simonis et al. (2010)	2.73	2.25	51.9 Simonis et al. (2010)	83.7
Jacobs et al. (2010)	2.55	0.16	98.8 NR *	93.9
Ma et al. (2010)	NR	0.29	100 Albrektsson and Isidor (1993) Roos et al. (1997)	100
Mertens et al. (2012)	3.26	0.3	100 Albrektsson et al. (1986)	100
Lops et al. (2012)	2.2	1.85	79.8 Albrektsson et al. (1986) Roos et al. (1997)	94.1
Gotfredsen (2012)	NR	0.75	NR Albrektsson and Isidor (1993)	100
Degidi et al. (2012)	2.54	1.95	34.9 Misch et al. (2008)	97.2
Deporter et al. (2012)	NR	1.21	95.5 NR *	95.5
Deporter et al. (2014)	NR	0.67	73.4 Albrektsson et al. (1986)	73.4
Ravald et al. (2013)	3.93	0.55	NR	95.1
Rocci et al. (2012)	NR	0.1	NR	91.1
Mangano et al. (2014)	NR	0.62	95.9 Zarb and Albrektsson (1998)	98.5
Adler et al. (2019)	NR	NR	NR	82.6

NR, not related; mm, millimeters; MPOM, mean marginal bone loss; MPS, mean probing depth; %, percentage. * The authors applied their own criteria of success or those of other authors (not related).

## Data Availability

The data that support the findings of this study are available on request from the corresponding author.
